# Livestock, methane, and climate change: The politics of global assessments

**DOI:** 10.1002/wcc.790

**Published:** 2022-05-27

**Authors:** Ian Scoones

**Affiliations:** ^1^ PASTRES Programme, Institute of Development Studies University of Sussex Brighton UK

**Keywords:** climate, conservation, global assessment, life‐cycle analysis, livestock, methane, politics

## Abstract

The relationship between livestock production and climate change is the subject of hot debate, with arguments for major shifts in diets and a reduction in livestock production. This Perspective examines how global assessments of livestock‐derived methane emissions are framed, identifying assumptions and data gaps that influence standard life‐cycle analysis approaches. These include inadequate data due to a focus on industrial not extensive systems; errors arising due to inappropriate emission factors being applied; questions of how global warming potentials are derived for different greenhouse gases and debates about what baselines are appropriate. The article argues for a holistic systems approach that takes account of diverse livestock systems—both intensive and extensive—including both positive and negative impacts. In particular, the potential benefits of extensive livestock systems are highlighted, including supporting livelihoods, providing high‐quality nutrition, enhancing biodiversity, protecting landscapes, and sequestering carbon. By failing to differentiate between livestock systems, global assessments may mislead. Inappropriate measurement, verification and reporting processes linked to global climate change policy may in turn result in interventions that can undermine the livelihoods of extensive livestock‐keepers in marginal areas, including mobile pastoralists. In the politics of global assessments, certain interests promote framings of the livestock‐climate challenge in favour of contained, intensive systems, and the conversion of extensive rangelands into conservation investments. Emerging from a narrow, aggregated scientific framing, global assessments therefore can have political consequences. A more disaggregated, nuanced approach is required if the future of food and climate change is to be effectively addressed.

This article is categorized under:

- Integrated Assessment of Climate Change > Assessing Climate Change in the Context of Other Issues
- Climate and Development > Social Justice and the Politics of Development

## Graphical abstract

Livestock at well, Isiolo, Kenya

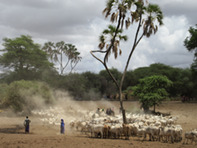

## 1 INTRODUCTION

The relationship between livestock production and greenhouse gas emissions is the subject of multiple global assessments and much public and policy commentary. Too often, this results in misunderstandings, rooted in a poor comprehension of both the impacts and benefits of different systems of livestock production, despite plentiful, comprehensive, evidence‐based reviews (e.g., Alibés et al., 2020; Herrero et al., 2016, 2009; Paul et al., 2020; Rivera‐Ferre et al., 2016). A generalized narrative frequently prevails, which argues for major shifts in diets to reduce meat and milk consumption and a reduction in livestock production worldwide, releasing land for conservation uses and rewilding. This article challenges this now widely held narrative, arguing for a more differentiated perspective, based on a more sophisticated approach to global assessments.

This is important since methane—the greenhouse gas emitted by ruminant livestock—has become the centre of recent climate mitigation debates (Reisinger et al., 2021). As a powerful “climate‐forcing” gas, methane has major effects on global warming, even though its lifetime in the atmosphere is short relative to carbon dioxide. Livestock production, together with gas pipelines, shale fracking, waste dumps and wet rice agriculture, is a significant emitter of methane.^1^ Reducing methane therefore is seen as a “quick win” for climate mitigation due to its significant influence on warming in the short term, and the Global Methane Pledge that commits to reducing methane by 30% by 2030 has over 100 countries as signatories.^2^

Efforts to reduce methane emissions will have major implications for livestock production globally, as systems of greenhouse gas measurement, verification, and climate emissions reporting are established. But which livestock, where? What are the uncertainties within the global scientific assessments central to framing mitigation policies? What assumptions and biases may distort, with what consequences? These are just some of the questions addressed in this article, which is based on the recent report, *Are Livestock Always Bad for the Planet? Rethinking the Protein Transition and Climate Change Debate* (Houzer & Scoones, 2021, https://pastres.org/livestock-report/).

## 2 DIVERSE LIVESTOCK, DIFFERENT EMISSIONS

The notion of the “livestock sector” presented in many global assessment reports is largely meaningless.^3^ There are hugely different livestock production systems in different parts of the world: from contained, industrial factory farming to extensive grazing on open rangelands. Along a long continuum, there are very different emission dynamics and so very different framings of and solutions to the methane mitigation challenge.

Extensive livestock producers make use of rangelands that cover over half the world's land surface, with many millions of producers tending everything from cattle and camels to goats and sheep to yaks, reindeer and llamas (ILRI et al., 2021). They are frequently marginalized economically and politically and are often seen as “backward” and “destructive” due to their mobile lifestyles. Yet extensive livestock producers, including pastoralists, make use of environments where conventional agriculture is impossible. They contribute to enhancing biodiversity, protecting ecosystem services, preserving cultures and landscapes and contributing high‐density protein and other nutrients to diets, often to those who need them most (Manzano et al., 2021). For obvious reasons, they are very different to large, industrial livestock production systems.

Lumping all livestock systems together in any analysis therefore makes little sense. There are different costs and benefits, different patterns of emissions, and different routes to mitigation. Yet, the way the science is framed in global assessments tends to aggregate and simplify. In order to see how these often‐inadvertent biases arise, we have to look at the science behind global assessments and how it is constructed. The choices of models, the units of assessment, the baselines used, and the styles of analysis all affect the results. The science is not simply “neutral,” but inevitably emerges through a social and political process where choices of framings and analysis strategies are made.

## 3 LIVESTOCK AND CLIMATE CHANGE: BIASES AND ASSUMPTIONS

The central tool in global assessments of livestock‐related climate impact is life‐cycle analysis. Such analyses assess all the inputs and outputs in a production system—sometimes stretching as far as transport, retail, and consumption—and so come up with a measure of emissions in relation to particular products. Such measures can then be aggregated and extrapolated across production units and geographies to derive an aggregate figure—the global contribution of the “sector” to total emissions.

In the case of livestock, a number of well‐known studies from the United Nations Food and Agriculture Organization have carried out such assessments, starting with the much‐debated *Livestock's Long Shadow* (Steinfeld et al., 2006; see also Glatzle, 2014), and more recently the report, *Tackling Climate Change through Livestock* (Gerber et al., 2013). The latter came up with the now much‐repeated “iconic fact” that 14.5% of total anthropogenic greenhouse emissions come from livestock.^4^

The aggregate emissions figures are presented in terms of carbon dioxide equivalents, meaning that all greenhouse gases, including methane, must be presented together. However, given the contrasting ways that gases behave in the atmosphere, they have very different “global warming potentials.” Much uncertainty exists around how to treat methane, for example, which has high warming potential in the short‐term, but decays rapidly. Some suggest that the effects of methane are overestimated using standard measures, and alternatives for assessing “global warming potential” have been proposed (Allen et al. 2016; Del Prado et al., 2021). Without going into the technical details, the point is that there is much uncertainty around the seemingly authoritative facts and figures about livestock's methane emissions.

Such uncertainties are compounded when the underlying emissions data on which they are based are examined. Inevitably, global assessments are estimates, but where does the data come from? In large part, livestock emissions data derive from respiratory chamber experiments on large, well‐bred animals, mostly in North America and Europe. Such experiments are used to derive emissions factors in national estimates reported to the Intergovernmental Panel on Climate Change (IPCC) and which subsequently are reflected in proposals for “Nationally Determined Commitments” for mitigation. However, a comprehensive review of life‐cycle assessments of food production systems highlighted that only 0.4% of these were from Africa (Clark & Tilman, 2017). When empirical studies are undertaken on local animals, very different results emerge to the standard emission estimates. In part, this is because such animals are smaller, but also because they are physiologically adapted and feed selectively on natural range under particular feeding regimes. As a result, such animals produce far fewer emissions than usually assumed in standard models (Goopy et al., 2021; ILRI, 2018; Ndung'u et al., 2019). While estimated emissions factors may be necessary while improved data emerges from such local studies, much caution must be applied to the results, since life‐cycle analyses may be substantially off the mark due to this chain of compounding uncertainties.

Yet, global assessments that feed into climate policy frequently rely on extrapolations from this type of life‐cycle analysis. A widely quoted example was published in *Science* and used information from an impressive 38,700 production units and 1600 processors as its data source (Poore & Nemecek, 2018). Extrapolating to a global level, the study claimed that reducing consumption of animal‐source foods and excluding animal production across 3.1 billion hectares (equivalent to a 19% reduction in arable land) would reduce greenhouse gas emissions by 49% (2018: 991). Without attending to the multiple qualifications and assumptions laid out in the Additional Materials, the media headlines that ensued condemned livestock production and urged major changes in diets, a message reinforced by the much‐debated EAT Lancet report (Willett et al., 2019). However, the *Science* study largely missed out on extensive systems, including as it did only “commercially viable” case studies. It relied on published data, which was mostly from industrial production in North America, Europe and some parts of Latin America and coastal China, and so created a distorted view, now replicated across public and policy debates.

Such biases in the data are in turn exacerbated by a narrow focus on emissions efficiencies per animal or per unit of product, without assessing the system as a whole, including the potential for sequestration. We still know very little about the carbon‐nitrogen dynamics in rangeland systems in different parts of the world (Garnett et al., 2017), but grasslands are a huge global store of carbon, notably in roots and soils (Dass et al., 2018); part of dynamic “open ecosystems” that have co‐evolved with natural herbivory, both domestic and wild, for millennia (Bond, 2019). Depending on the state of such ecosystems, grazing animals can add to such stocks (Conant et al., 2017; McSherry & Ritchie, 2013). This is especially the case if manure is deposited and incorporated across wide areas, as with mobile systems. More comprehensive life‐cycle assessments of mobile, extensive livestock production shows how, if such sequestration potentials are accounted for, then such systems can be in carbon balance, even being net positive under some conditions.^5^

All ruminants produce methane and so have impacts on global warming, but in global assessments for climate change mitigation we are interested in those that are “additional.” This means thinking about what the appropriate baseline is (Manzano & White, 2019). Emissions from industrial systems are clearly additional to a natural baseline. Animal‐derived emissions are added to by those from feed imports, concentrated waste deposition and infrastructure investments. However, an extensive system on an open rangeland may not in fact be additional to a natural baseline, where the same environment was previously occupied by other grazing herbivores. Assessments of emissions from wild animals, as well as termites, show how baseline emissions may be high; perhaps as high as the extensive livestock systems that replace them (Hristov, 2012). Differentiating between production systems in relation to their baselines and calculations of “additionality” is thus vitally important.

All this points to the need for a “systems” approach to life‐cycle assessments, avoiding the narrow focus on emissions per animal or unit of product as is conventionally applied. Instead, a more holistic assessment should encompass both emission impacts—including from production, inputs, waste, infrastructure, transport, and so on—and environmental benefits—including carbon/nitrogen sequestration, but also improvements in biodiversity, ecosystem services, landscape values, and so on. Such estimates in turn must take account of the actual animals involved, without imposing artificial emission factor estimates and, furthermore, assessments must be in relation to a realistic baseline, depending on what livestock are replacing. In turn, a wider social and economic assessment should evaluate the impacts of different livelihood options, examining, for example, how different systems provide both affordable and high‐quality animal products. Such analyses would then encompass the trade‐offs between cheap products versus high‐quality nutrition, and the meeting of particular dietary needs, especially of those who are nutritionally vulnerable, including younger people and pregnant or nursing women (e.g., Adesogan et al., 2020; Beal et al., 2021; Iannotti et al., 2021; Moughan, 2021).

There are of course many intersecting uncertainties and multiple trade‐offs inherent in such a systems analysis, but a mature, deliberative discussion of climate change mitigation options and the future of food requires engaging with uncertainties and addressing trade‐offs. Rushing to premature and distorting conclusions with the search for iconic, media‐friendly figures and associated targets can be highly damaging.

## 4 SCIENCE AND GLOBAL ASSESSMENTS: FRAMING THE DEBATE

How assessments are framed, what models are deployed, which data are used, and what baselines are applied therefore make a big difference. There is an important political economy in “which data count” in global assessments, and so in climate change policymaking. Despite the assured proclamations, amplified in the media and those campaigning for certain “solutions,” there are multiple, compounding uncertainties that impinge on any assessment. This of course is not an argument for doing nothing. But it is an argument for being more circumspect about generalized recommendations and being more careful in the analysis before leaping to conclusions that all livestock are bad for the planet everywhere, or that we must all change our diet.

Simple policy narratives run well in media headlines. For example, the *Science* paper mentioned earlier (Poore & Nemecek, 2018) has been repeatedly used, with the UK *Guardian* newspaper running the headline “avoiding meat and dairy is the single biggest way to reduce your impact on earth.”^6^ In the same way, the well‐respected data visualization site, Our *World in Data*, makes use of this same analysis in many of its curated graphs, arguing for shifts in diet and changing livestock systems.^7^ Looking for a clear campaign position, messages may essentialize and simplify, but may also distort with damaging consequences. The oft‐repeated argument that cows are just as bad as cars in terms of climate impacts misses many important nuances; not least that methane and carbon dioxide have very different impacts in the atmosphere, and the fact that emissions figures for livestock include both direct and indirect emissions (across the whole life cycle), while those for transport cover only direct emissions.^8^

The IPCC's AR6 Working Group 1 report focused a lot on methane and offered some nuanced, while sobering, assessment.^9^ However, most journalists will not wade through the dense, scientific text and will revert to the shorter summaries. Media headlines and campaign positions inevitably simplify. This would not matter if there were not material consequences. While most journalists and campaigners in environmental organizations will argue that their focus is on industrialized meat and dairy production, where most of the damage occurs, this is not always clear. The consequences of having only one form of aggregate assessment or a singular figure is that, in the global mechanisms designed to address climate change, a differentiated perspective is lost. Standardized measures are applied to come up with Nationally Determined Commitments presented as part of mitigation plans and so become part‐and‐parcel of agreed verification and reporting systems.

Poor quality data, lack of analysis capacity and inappropriate assessment models, inadequately applied, may end up with poor diagnoses that fail to distinguish between different livestock production systems. The ability to respond to the huge requirements of national climate impact assessment and reporting on mitigation options under UNFCCC (UN Framework Convention on Climate Change) procedures is especially challenging for under‐resourced officials in developing country environment ministries. It is of course much easier to pluck a number from an existing analysis and plug it into a very rough estimate for national emissions than to try and work it out from scratch, with virtually non‐existent, locally‐grounded data. As a result, errors and assumptions are accumulated and multiplied, resulting in persistent biases in aggregate global assessments of the climate impacts of livestock and distortions in debates about the future of food.

## 5 POLITICS AND INTERESTS

Global assessment processes are therefore inevitably political, favouring some interests over others. This may not be the result of any deliberate conspiracy, but the consequence of the processes of aggregation, simplification and the limits in capacity for data collection and analysis.

The systematic biases against extensive livestock producers, most notably pastoralists, are evident however. This has very direct consequences, with major injustices arising as fingers of blame are pointed inappropriately (García‐Dory et al., 2021). Aggregation and lack of differentiation may also get others off the hook. Industrial meat and dairy production results in major negative environmental impacts; not only in terms of high levels of methane emissions, but also in terms of fossil fuel consumption, feed imports, water pollution, and the costs of transport and infrastructure (Weis, 2013).^10^

Yet, advocates of industrialized, contained livestock production will consistently point to the high per animal or unit of product emissions from animals grazed extensively on rough forage, and the potential for mitigating this through intensification, through feed additives, vaccines, or other technological measures (cf. Beauchemin et al., 2020). This argument is presented in terms of improving “efficiency” and “modernizing” production; doing away with what are seen as wasteful and inefficient extensive systems. As we have seen this is a very selective view, failing to look at the full range of both impacts and benefits as required by a more holistic, systems view. But it nevertheless serves certain corporate farming interests that make profits from “big meat and dairy,” and feeds into a narrative of technological modernization with a narrow view of efficiency at its core (Ajl & Wallace, 2021).

What is “modern” or “efficient” depends on your perspective of course. Many have made the case that in making use of highly variable, low‐productivity rangelands and converting grassland into high‐quality protein and other nutrients, mobile pastoralists—cast as “backward” by industrial system advocates—are actually highly modern and efficient (Krätli, 2015). Arguments that project extensive livestock producers as “climate villains” therefore are political positions, framed in terms of supposedly technical arguments, ones that of course are never neutral but come with many cultural, economic, and social assumptions.

Corporate backers of intensive livestock production have some unexpected allies, including those who argue for the reduction in extensive livestock to create space for biodiversity conservation. Adopting similar rhetoric about the inefficiency and destructiveness of extensive livestock production, some argue for protecting half the earth for conservation and biodiversity protection (without people and their domestic animals) and reserving the other half for more intensive use, including for food (Wilson, 2016).^11^ Those offering often over‐hyped promises of industrially‐manufactured “cultured meats” and plant‐based “alternative proteins” (Sexton et al., 2019) frequently adopt a similar narrative, aiming to release land from extensive use—for example for “rewilding”—while intensifying food production elsewhere (Dinerstein et al., 2020; Folberth et al., 2020). Such views are promulgated despite evidence that certain types of careful grazing can enhance biodiversity and reduce the risks of wildfires and other catastrophic, climate‐damaging losses. As a result, such analyses usually proceed without a full evaluation of the costs of producing protein through industrial intensification, whether from animals, plants, or cultured alternatives (Houzer & Scoones, 2021; Manzano et al., 2021).

An unlikely coalition of scientists, policy‐makers, environmental campaigners, journalists, diet‐change activists, and those backing industrial production of protein has emerged, focused on technological solutions to climate mitigation. Inadequate data and inappropriate analysis supports such positions, while condemning and vilifying extensive livestock production. Yet, due to a lack of voice and influence in global assessments, compounded by multiple scientific uncertainties, inappropriate assumptions, and data gaps, injustices arise for extensive livestock producers, particularly pastoralists. This in turn undermines effective global debate on the future of food and climate change.

## 6 CONCLUSION

What then is the way forward that allows for more balanced global assessments of livestock's contributions to emissions and so a more informed debate that challenges the misleading assumptions and redresses the political inequalities in current discourse?

- First, there is a need for differentiation between production systems. It is the material conditions of production—and so the relations of capital, labour and environment—that matter, not the products. Meat and milk are very different depending on how they are produced.
- Second, any assessment must adopt an integrated, holistic systems approach, looking at all inputs and outputs and all costs and benefits for the whole system, while examining the uncertainties influencing conclusions and the trade‐offs that arise.
- Third, any analysis must interrogate the different framings at play, examining what is wanted from the system, taking account of the values, ethical positions, and material needs of different actors. Livestock systems look very different from different standpoints.
- Fourth, much more dialogue and deliberation about what the priorities are is needed—between say the cost of food and its impacts on the environment and wider livelihoods. This means surfacing the uncertainties and assumptions at the centre of standard life‐cycle analyses, and so questioning the modeling approaches, as well as the appropriateness of the data for different settings.
- Fifth, more research must look at, for example, emission factors of different types of animals in different agroecosystems and the patterns and potentials of carbon/nitrogen sequestration in open rangelands. In addition, different ways of conducting assessments are required, involving more diverse actors in engaged deliberation about how model parameters are chosen and figures derived, interrogating the framing of both problems and solutions.
- Sixth, all this suggests much more attention to the political economy of assessments. Some outcomes may benefit narrow interests and exclude others, imposing injustices in ways that undermine livelihoods, generate poverty, and reduce the opportunities of the often already marginalized. Addressing whose knowledge counts, who wins and who loses, and whose interests prevail is therefore vital.

In sum, creating space for the voice of pastoralists and other extensive livestock producers in global debates about the future of food and the climate is essential. This will not only improve the science, but also the possibilities of addressing the major challenges of our time in a more balanced and equitable way.

## FUNDING INFORMATION

The writing of this article was supported by a European Research Council Advanced Grant (740342).

## CONFLICT OF INTEREST

The author has declared no conflicts of interest for this article.

## RELATED WIREs ARTICLES

Farmers fighting climate change—from victims to agents in subsistence livelihoods

Re‐framing the climate change debate in the livestock sector: mitigation and adaptation options

## Data Availability

Data sharing is not applicable to this article as no new data were created or analysed.

## Endnotes

^1^
There were over 650 mentions of “methane” in the full WG1 AR6 report, see https://www.ipcc.ch/report/ar6/wg1/.

^2^

https://ec.europa.eu/commission/presscorner/detail/en/STATEMENT_21_5766

^3^
The IPCC's AR6 WG3 report on mitigation does acknowledge the difference between intensive and extensive systems, but also slips into generalizations about ‘livestock’ as a whole in discussing mitigation options; see https://www.ipcc.ch/report/ar6/wg3/.

^4^
Including both direct and indirect emissions across the life cycle, amounting to 7.1 gigatons of CO_2_ equivalent per year. Direct livestock emissions account for 5% of the total, according to the FAO estimate.

^5^
See for example studies in Sardinia, Italy (Arca et al., 2021), Amdo Tibet, China (Zhuang & Li, 2017) and the Ferlo, Senegal (Assouma et al., 2019); see also Houzer and Scoones (2021, pp. 38–46).

^6^

https://www.theguardian.com/environment/2018/may/31/avoiding-meat-and-dairy-is-single-biggest-way-to-reduce-your-impact-on-earth;

^7^

https://ourworldindata.org/environmental-impacts-of-food

^8^
See “Cars or Livestock: Which Contribute More to Climate Change?,” www.news.trust.org/item/20180918083629-d2wf0and https://www.cgiar.org/news-events/news/fao-common-flawed-comparisons-greenhouse-gas-emissions-livestock-transport/.

^9^

https://www.ipcc.ch/report/ar6/wg1/

^10^

https://friendsoftheearth.eu/wp-content/uploads/2021/09/MeatAtlas2021_final_web.pdf

^11^
For example, https://www.campaignfornature.org/Background or https://www.weforum.org/agenda/archive/trillion-trees
